# Prevalence, Awareness and Control of Hypertension in Uganda

**DOI:** 10.1371/journal.pone.0062236

**Published:** 2013-04-17

**Authors:** Geofrey Musinguzi, Fred Nuwaha

**Affiliations:** 1 Department of Disease Control and Environmental Health, School of Public Health, College of Health Sciences, Makerere University, Kampala, Uganda; The University of Manchester, United Kingdom

## Abstract

**Background:**

Prevention and control of hypertension are critical in reducing morbidity and mortality attributable to cardiovascular diseases. Awareness of hypertension is a pre-condition for control and prevention. This study estimated the proportion of adults who were hypertensive, were aware of their hypertension and those that achieved adequate control.

**Methods:**

We conducted a community based cross sectional survey among people≥15 years in Buikwe and Mukono districts of Uganda. People had their blood pressure measured and were interviewed about their social-demographic characteristics. Hypertension was defined as systolic blood pressure ≥140 mmHg, or diastolic blood pressure ≥90 mmHg, or previous diagnosis of hypertension. Participants were classified as hypertensive aware if they reported that they had previously been informed by a health professional that they had hypertension. Control of hypertension among those aware was if systolic blood pressure was <140 mmHg and diastolic blood pressure was <90 mmHg.

**Results:**

The age standardized prevalence of hypertension was 27.2% (95% CI 25.9–28.5) similar among females (27.7%) and males (26.4%). Prevalence increased linearly with age, and age effect was more marked among females. Among the hypertensive participants, awareness was 28.2% (95% CI 25.4–31.0) higher among females (37.0%) compared to males (12.4%). Only 9.4% (95% CI 7.5–11.1) of all hypertensive participants were controlled. Control was higher among females (13.2%) compared to males (2.5%).

**Conclusion:**

More than a quarter of the adult population had hypertension but awareness and control was very low. Measures are needed to enhance control, awareness and prevention of hypertension.

## Introduction

The epidemiological transition with increasing prevalence of chronic non-communicable diseases (NCDs) is already underway in sub-Saharan Africa [1], [2], [3], [4],[5], [6], [7], [8], [9], [10]–[11], [12]including Uganda[5], [6], [7], [12]. The commonest NCDs experienced during this early stage of the epidemiological transition are hypertension [6], [7], [10] and cerebral vascular accidents (strokes) with strokes in sub-Saharan Africa mainly attributable to uncontrolled hypertension [8], [9]–[10]. In Uganda, hypertension is the most reported NCD [6], [7]. As observed in most countries of sub-Saharan Africa, there is still lack of awareness about the growing problem of NCDs, which, unfortunately, is often coupled with the absence of a clear policy framework for prevention and management [6], [7], [10]. Moreover, in the low income countries hypertension is increasingly affecting young people less than 50 years, many of whom die prematurely as compared to the developed countries [8], [9], [10], [11].

Studies examining awareness and control of hypertension in Uganda and SSA are scarce, despite the reported increase in the prevalence of hypertension in the region [1], [2], [3], [4]. Information on awareness and control of hypertension is needed for planning effective control strategies and for resource mobilization [6], [7]. This study was undertaken to estimate awareness and control of hypertension in Buikwe and Mukono districts of Uganda with a view of suggesting measures for remedial action.

## Methods

### Study population and setting

The study was conducted among adults 15 years and above in urban and rural communities of Buikwe and Mukono districts of Uganda. Buikwe was carved out of Mukono district in 2009. These two districts neighbour Lake Victoria and lie between the two biggest urban areas of Jinja and Kampala (the capital city). Together the two districts have a population of over 1,000,000 people (projected from the 2002 national census at annual growth rate of 4%). About 75% of the population are rural dwellers whose main occupation is agriculture. Mukono and Buikwe districts were chosen because of their strategic location between two major urban centers of Kampala and Jinja as well as the likelihood of having a large section of urban dwellers.

### Study design

This was a community based cross sectional study where data was collected through interviews and taking of physical measurements. The tool used for data collection was adapted from the World Health Organisation (WHO) stepwise approach to chronic disease risk factors surveillance (STEPS) [13].

### Data collection

The questionnaire enlisted social demographic and behaviour variables such as age, sex, place of residence, level of education, marital status, alcohol and tobacco smoking. Physical measurements conducted included the height, weight, and blood pressure. In addition a medical history was elicited regarding whether the individual had ever had their blood pressure measured, whether they have ever been told by a health professional that they have hypertension and whether they were receiving treatment for hypertension in the previous two weeks preceding the survey. The questionnaire administration and blood pressure measurements were carried out by trained research assistants from July through September of 2012 in people's homes. Data collection was conducted every day of the week including weekends and evenings when men in urban settings were more likely to be found at home.

### Blood pressure measurement

Blood pressure (BP) was measured on a single occasion using automated digital blood pressure monitor, model LD7 with appropriate cuff sizes. Three blood pressure measurements (at least 1 minute apart) were taken after 5 minutes rest with the participant seated, using a calibrated automated digital BP machine. For each participant's blood pressure, the mean of the three values was calculated to estimate their blood pressure. A participant was classified as being hypertensive if their mean systolic BP was 140 mmHg or higher, or if their average diastolic BP was 90 mmHg or higher, or if they were on anti-hypertensive treatment in the two weeks preceding the study or if they had previously been told by a health professional that they had hypertension or the combination of the above. Participants were classified as hypertensive aware if they reported that they had previously been informed by health professional that they had hypertension. Hypertensive aware participants were classified as being on treatment if they reported current use of drugs prescribed by a health professional which they had taken within the past two weeks prior to the study. Control was defined as systolic blood pressure <140 mmHg and diastolic blood pressure <90 mmHg among people aware of their hypertension.

### Sampling

A random sample of 111 villages stratified by residence status (rural or urban) was generated using a sampling frame of the 2002 Uganda National Census and Housing enumeration areas available from the Uganda Bureau of Statistics [14]. An urban area was defined as a town with a population of at least 10,000 inhabitants according to the Uganda 2002 national census [14]. From each village, 30 households were systematically sampled beginning from a central point of the village and individuals aged 15 years and above in a household were enrolled into the study as long as they provided consent and were not within the exclusion criteria such as the pregnant women. Pregnant women were excluded because of the role of pregnancy in modifying blood pressure.

### Ethics statement

The study was approved by Makerere University School of Public Health institutional review board and the Uganda National Council of Science of Technology. Written informed consent was obtained from adult participants. For participants below the age of 18, assent and written informed consent were obtained from minors and their parents/guardians respectively. People diagnosed with hypertension were referred to health units.

### Analysis

Data that was collected by trained research assistants from July to September 2012 was field edited and checked for consistencies on a daily basis after each working day. Consequently, the data was entered in EpiData 3.02 (http://www.epidata.dk), cleaned and exported using Stata Transfer to STATA 10.0 (Texas, USA) for analysis. The prevalence of hypertension was age standardized using World Health Organisation (WHO) world population for people aged 15 years and above. In order to adjust for clustering, robust standard errors and cluster option in the STATA 10.0 software were utilized. We conducted stratified analysis by sex and classified outcomes variables according to residence, age, marital status, school attendance, alcohol consumption, tobacco smoking and Body Mass Index (BMI). To identify independent predictors of being hypertensive; age, residence, level of education, marital status, tobacco smoking, alcohol use and BMI were controlled for each other using multivariable logistic regression. The crude odds ratios (COR) were compared to adjusted odds ratios (AOR) and their 95% confidence intervals (CI). Awareness and control of hypertension was assessed by calculating proportions with their CI. For awareness the number of those who were aware was divided by those who were hypertensive. For control the numerator was those controlled and the denominators were those hypertensive, aware or on treatment. Significance for proportions was tested using a two tailed Pearson's chi-square test with Yates's collection. Linear trends between hypertension and age, education and BMI were assessed with Cochran-Armitage chi-square for linear trend. For all tests, a p-value of <0.05 was taken as statistically significant.

## Results

Four thousand eight hundred and eighteen people (3183 women, 1635 men) were found at home and consented to participate in the study. Of those who consented 222 (7% of the women) were excluded because they reported being pregnant. Thirty three of the 4596 study participants (12 men and 21 women) were excluded from analysis because of missing data. Of the 4563 analysed 64.5% where females and 35.5% were males. More females were included because they were more likely to be found at home compared to men. No more data was collected about people not at home at the time of the survey.

### Characteristics of the studied population

Table 1 illustrates comparisons of selected characteristics stratified by sex. The mean age was higher among males compared to females [36.2 (16.6) versus 34.1 (14.9) years P<0.001]. The majority of study participants were in the age range 15–24 (31.9%), followed by the 25–34 year olds (25.3%). A higher proportion of females were aged 25–34 years whereas higher proportion of males compared to females were in the age groups above 45 years (table 1) About two thirds of the participants resided in rural areas and this proportion was the same among females and males. It can be depicted from table 1 that 12.1% of the study participants never attended school and of those who reported attending school, majority completed primary education (44.1%). Proportions across sex show that non school attendance was higher among females (13.8%) compared to males (9.1%) and proportion of primary completion was high among females (45.3%) than males (41.9%). Secondary school and tertiary school attendance was significantly higher among males compared to females. The ethnic distribution was highly diverse representing almost every tribe in Uganda but with the Baganda contributing the biggest percentage (50%). More than a half (57.8%) of the study population was married and the proportion of widowed respondents was more than four times among females (9.8%) compared to males (2.3%). The proportion of the never married was twice among males (32.2%) compared to females (16.4%). Being divorced or separated was significantly higher among females compared to males. More than 60% of the study population earned less than $200 every month. Men were more likely to be current consumers of alcohol and smokers compared to females. Obesity and overweight were higher in females compared to males whereas underweight was lower in females compared to males (table 1).

**Table 1:** Characteristics of the study respondents.

Variable	Total (N = 4563)	Female (N = 2940)	Male (N = 1623)	P-value
	n(%)	n(%)	n (%)	
Age in years				
15–24	1455 (31.9)	951 (32.3)	504 (31.1)	
25–34	1155 (25.3)	784 (26.7)	371 (22.9))	
35–44	773 (16.9)	508 (17.3)	265 (16.3)	
45–54	591 (13.0)	355 (12.1)	236 (14.5)	
55–64	309 (6.8)	180 (6.1)	129 (8.0)	
65+	280 (6.1)	162 (5.5)	118 (7.2)	<0.001
Education level				
None	554 (12.1)	406 (13.8)	147 (9.1)	
Primary	2012 (44.1)	1332 (45.3)	680 (41.9)	
Secondary	1625 (35.6)	1003 (30.7)	502 (38.3)	
Tertiary	372 (8.2)	199 (10.2)	293 (18.1)	<0.001
Place of residence				
Urban	1520 (33.3)	996 (33.9)	524 (32.3)	
Rural	3043 (66.7)	1944 (66.1)	1099 (67.7)	0.28
Marital status				
Never married	1006 (22.1)	483 (16.4)	523 (32.2)	
Currently married	2638 (57.8)	1723 (58.6)	915 (56.4)	
Divorced/separated	592 (13.0)	446 (15.2)	146 (9.0)	
Widowed	326 (7.1)	288 (9.8)	39 (2.3)	<0.001
Current smoker				
Yes	325 (7.1)	71 (2.4)	254 (15.7)	
No	4238 (92.9)	2869 (97.6)	1369 (84.3)	<0.001
Currently drinks alcohol				
Yes	1093 (24.0)	547 (18.6)	546 (33.3)	
No	3470 (76.0)	2393 (81.4)	1077 (66.4)	<0.001
Body mass index				
Under weight	588 (12.9)	323 (11.0)	265 (16.3)	
Normal	3074 (67.4)	1849 (63.0)	1225 (75.4)	
Over weight	616 (13.5)	513 (17.4)	103 (6.4)	
Obese	285 (6.2)	255 (8.6)	30 (1.8)	<0.001

### Prevalence of hypertension

The overall prevalence of hypertension in our sample was 21.8%. The age adjusted prevalence was 27.2% and was similar among women and men [Female 27.7% (95% CI 26.1–29.3) and men 26.4% (95% CI 24.3–28.5)]. The prevalence of hypertension increased linearly with age overall (Chi-square for linear trend = 686.9, P<0.001) and the increase was more marked among women (table 2). Young women less than 35 years were less likely to be hypertensive compared to young men of the same age group. Above 35 years the trend reversed with more women than men likely to be hypertensive. Within the 15–24 years old, the prevalence of hypertension among men (11.6%) almost doubled that of women (6.5%). Hypertension was higher (37.5%) among men in urban areas compared to those in rural areas (19.6%). In the female category, there was no rural urban gap (rural: 21.7% versus 21.7%). Meanwhile, the prevalence of hypertension among rural women was not higher compared to rural men (women: 21.7% versus men 19.6%). In the urban strata hypertension was less common among women (21.7%) compared to men (37.5%). Not attending school was associated with higher prevalence of hypertension in both sex categories. Among those who never attended school, the prevalence was higher (41.8%) among females than men (27.9%) and within the women; the prevalence was about three times among those who never attended school compared to those who had attained tertiary education (41.8% versus 14.1%). The prevalence of hypertension appears to decrease with level of education among the women (Chi-square for linear trend = 122.2, P<0.001) but not among men (Chi-square for linear trend = 1.7, P = 0.19). The prevalence of hypertension was highest among widowed women (49.5%) followed by those who were divorced or separated (30.5%) then by the currently married (18.2%) and finally prevalence of hypertension was lowest among the never married (9.7%). Unlike the women hypertension was the same among those currently married (26.8%), separated or divorced (27.4%) and widowed (28.9%) but was lowest among the never married men (12.6%). Among both females and males, hypertension was higher among people who smoked, drank alcohol and among the obese as well as the overweight (table 2).

**Table 2:** Prevalence of hypertension among females and males by age, education status, place of residence, marital status, smoking, use of alcohol and body mass index.

Variable	Total (N = 4563)		Female (N = 2940)		Male (N = 1623)	
	%	CI	%	CI	%	CI
Overall	21.8	20.6–23.0	21.7	20.2–23.2	22.3	20.3–24.3
Age in years						
15–24	8.3	6.8–9.7	6.5	5.1–8.3	11.7	9.1–14.8
25–34	13.6	11.6–15.5	12.1	10.0–14.6	16.7	13.4–20.9
35–44	25.1	22.0–28.1	26.1	22.5–30.1	23.5	18.9–29.0
45–54	35.5	31.6–39.3	39.1	34.1–44.3	30.6	25.1–36.8
55–64	50.2	44.5–55.7	59.1	51.7–66.1	39.5	31.5–48.2
65+	58.6	52.5–64.6***	68.1	59.9–75.3***	47.9	39.0–56.9***
Education level						
None	37.9	33.8–41.9	41.8	37.1–46.7	27.9	21.3–35.6
Primary	22.0	20.1–23.7	21.5	19.3–23.8	23.1	20.1–26.4
Secondary	17.0	15.1–11.8	15.5	13.2–18.0	19.7	16.4–23.4
Tertiary	18.5	14.5–25.5***	14.1	10.6–18.5***	22.2	17.8–27.3 *ns*
Place of residence						
Urban	23.6	21.4–25.7	21.7	19.3–23.7	37.5	32.0–42.3
Rural	21.0	19.5–22.4*	21.7	19.9–23.7ns	19.6	17.3–22.2**
Marital status						
Never	11.2	9.2–13.1	9.7	7.4–12.7	12.6	10.0–14.9
Married	22.1	19.5–22.6	18.2	16.4–20.1	26.8	24.0–29.8
Divorced/separated	29.7	26.0–33.4	30.5	26.4–34.9	27.4	20.8–35.2
Widowed	46.5	41.0–51.8***	49.5	43.6–55.3***	28.9	17.0–44.9***
Current smoker						
Yes	28.6	23.6–33.5	40.0	28.1–53.2	26.4	21.0–32.7
No	21.3	20.0–22.5**	21.3	20.0–22.9**	21.7	19.6–23.9ns
Current consumption of alcohol						
Yes	26.8	24.9–28.5	27	23.4–30.9	29.0	25.3–32.9
No	17.2	15.6–18.7***	20.5	18.9–22.1**	19.0	16.7–21.4***
Body mass index						
Normal	18.7	17.3–20.1	17.2	15.6–19.0	21.0	19.0–23.3
Under weight	21.9	18.5–25.2	27.2	21.8–33.3	19.1	14.1–25.4
Over weight	29.2	25.6–32.8	28.0	24.3–32.1	36.8	28.2–44.6
Obese	41.2	35.2–47.2***	40.2	34.0–46.6***	63.6	42.7–80.3***

CI 95% Confidence interval

ns p>0.05; * p<0.05; ** p<0.01; *** p<0.001

In a multivariable logistic regression model that controlled for sex, age, education level, residence, marital status, tobacco smoking, alcohol consumption and BMI, the independent predictors of being hypertensive were age, residing in an urban area and being overweight or obese (table 3).

**Table 3:** Independent predictors of hypertension.

Variable	COR (CI)	AOR (CI)
Sex		
Male	Reference	Reference
Female	1.0 (0.8–1.1)	0.9(0.8–1.1)
Age in years		
15–24	Reference	Reference
25–34	1.7 (1.3–2.2)	1.5 (1.2–1.9)
35–44	3.6 (2.9–4.7)	3.0 (2.3–4.4)
45–54	6.0 (4.7–7.8)	5.3 (4.1–6.9)
55–64	11.0 (8.3–14.8)	10.5 (7.5–14.3)
65+	15.6 (11.4–21.3)	17.5 (11.9–26.3)
Educational level		
None	Reference	Reference
Primary	0.4 (0.3–0.5)	0.7 (0.5–1.0)
Secondary	0.3 (0.2–0.4)	0.9 (0.9–1.3)
Tertiary	0.3 (0.2–0.5)	0.5 (0.7–1.3)
Place of residence		
Rural	Reference	Reference
Urban	1.2 (1.1–1.4)	1.5 (1.2–1.8)
Current smoker		
No	Reference	Reference
Yes	1.4 (1.1–1.9)	0.9 (0.7–1.3)
Currently drinks alcohol		
No	Reference	
Yes	1.8 (1.5–2.0)	1.2 (0.9–1.4)
Marital status		
Never married	Reference	Reference
Married	2.1(1.7–2.6)	1.3 (0.8–1.4)
Separated/divorced	3.3 (2.5–4.5)	1.2 (0.9–1.2)
Widowed	6.8 (5.1–9.2)	1.3 (0.9–1.1)
Body mass index		
Normal	Reference	Reference)
Underweight	1.2 (1.0–1.5)	0.8 (0.6–1.1)
Over weight	1.7 (1.5–2.2)	1.5 (1.2–1.9)
Obese	3.0 (2.3–4.0)	2.3 (1.8–3.2)

COR Crude odds ratio AOR Adjusted odds ratio

CI 95% Confidence interval

### Awareness and control hypertension

Overall 1268 (36.5%, CI 35.1–37.9%) of the study participants had ever had their blood pressure measured. Females were more likely to have ever had their blood pressure measured compared to males [women 36.5% versus men 12.0%]. Among the 997 people that had hypertension 283 (28.2%, CI 25.4–31.0) were aware that they have the disease and awareness was high among the women compared to males [women 37.4% versus men 12.4%]. Of those aware of the disease 142 (51.6% CI 45.7–57.4) were on treatment within two weeks of the interview and 93 (33.1%, CI 27.6–38.6%) had achieved control of the disease. More men (62.2%) than women (48.7%) were likely to be on treatment but the difference was not statistically significant (table 4). Control among all those with hypertension was significantly high among females (13.2%) than males (2.5%). Control among those on treatment was achieved by 52 (35.9%, 28.1–43.7) whereas among all people with hypertension only 93 (9.3%, CI 7.5–11.1) were controlled. The details of awareness and control stratified by sex are shown in table 4.

**Table 4:** Awareness and control of hypertension among females and males.

Variable	Female			Male			P value
	n/N	%	CI	n/N	%	CI	
							
BP ever measured	1073/2940	36.5	33.6–39.4	195/1623	12	10.4–13.6	<0.001
Aware of hypertension	238/635	37.4	33.7–41.2	45/362	12.4	9.0–15.8	<0.001
							
On treatment	114/234	48.7	43.5–56.3	28/45	62.2	46.8–75.0	0.097
Control among hypertensive	84/635	13.2	10.6–15.8	9/362	2.5	0.9–4.1	<0.001
Control among aware	84/238	35.2	29.1–41.4	9/45	20.0	8.1–31.8	0.045
Control among those on treatment	35/114	30.7	22.1–39.2	4/28	14.2	1.0–27.6	0.08

n: number with condition (numerator) N: denominator

Awareness increased linearly with age from 1.7% among those aged 15–24 to 38% among those aged more than 65 years (Chi-square for linear trend = 68.3, P<0.001). However, awareness was not affected by level of education (Chi-square = 5.5, 3df, P = 0.14). Although awareness was high in rural areas (29.7%) than urban areas (24.6%), this difference was not statistically significant (Chi-square = 5.5; 1df, P = 0.076).

## Discussion

In a population based survey of adults 15 years and above conducted in the districts of Mukono and Buikwe in Uganda hypertension was very common with more than one in five of the people affected. The finding is consistent with studies conducted in other parts of the country as well as in sub-Saharan Africa which show the prevalence of high blood pressure ranging from 20% to 50% [1], [2], [3], [4], [5], [6], [7], [12]. A comparison with these previous studies is however, best interpreted with caution as the studies used different age groups. In addition, our study revealed a high prevalence of hypertension among individuals 15–24 years, with evidence of hypertension being higher among males compared to females. This finding reinforces evidence that hypertension is increasingly affecting young people in the low income countries [10]. As observed elsewhere the prevalence of hypertension increases with increasing age [1],[2],[3], [4], [5], [6], [7] and the increase is was more marked among women compared to men [1], [4], [6], [11]. In our study the prevalence of hypertension was higher among urban residents and among those who are overweight as well as the obese. These observations suggest that demographic transition and urbanization are major determinants of hypertension and as the life expectancy increases in low income countries and people migrate to urban areas, the burden of hypertension and other cardiovascular diseases will increase [8], [9], [10], [15,[16], [17]. Overweight and obesity are attributed to changes in dietary and physical activity patterns which are often the result of urbanisation and societal changes attributed to development and lack of supportive policies in health and other related sectors [18], [20], [21], [22].

Despite the high prevalence of hypertension, awareness of the problem was very low at less than 30 percent. The low awareness could be explained by our study findings that showed only 27.8% of the population to have had their blood pressure ever measured. Awareness of hypertension largely depends on the capacity of the health system to provide diagnostic services for hypertension to the general population [4], [22]. Unfortunately, the healthcare system in Uganda is largely constrained by communicable diseases and NCDs have not received the attention they deserve [6], [7]. As expected awareness was much better among women compared to men and this trend has been observed in high as well as low income countries. A plausible explanation that have been suggested for this trend is the more frequent contact of women with health services because of maternal and child health programs [4], [22]. However, hypertension is largely asymptomatic and in order to increase awareness, there is need to screen all adults at an appropriate opportunity when they get in contact with health system. Additionally, outreach and community programs for detection of hypertension may have to be developed and tested as has been successfully done with other asymptomatic diseases [23], [24].

Among the people with hypertension less than 10% were controlled. Reasons for this very low level of control is that the majority of people with hypertension are not aware and even among those aware less than a half were receiving treatment. However, even among those receiving treatment only one in three had achieved control. A worrying global trend is that very low levels for control of hypertension are widespread in both low and high income countries [4], [25], [26]. In a recent systematic review of 44 articles from 35 countries the authors found no significant cross-sectional differences between developed and developing countries in hypertension indices. In terms of control among all hypertensive, 10.8% of the men had adequate control in high income countries compared to 9.8% in low income countries. Among women only 17.3% of all people with hypertension achieve control compared to 16.2% in low income countries [4]. Thus although effective control of hypertension largely depend on quantity and quality of the healthcare system [22] these data suggest that adequate control at a population level is extremely difficult in practice. Indeed in Uganda and most of Sub-Saharan Africa where health care is grossly inadequate with low levels of health staffing and a chronic under supply of medicines it is almost an insurmountable task to achieve control of emerging NCDs such as hypertension. In addition to the NCDs infectious diseases are still prevalent in the region [6], [7], [27].

Advantages of the present study were the large sample size stratified by sex and the use of a standardized protocol. However, the sample was not strictly representative, as more women than men were found at home and therefore more likely to be involved in the study (see table S1 comparing the population structure of our sample with the 2002 national census). This limitation makes it difficult to generalise our findings to other populations. Prevalence of hypertension may have been overestimated and control of the condition underestimated because the three blood pressure measurements were performed on one occasion only. This was done for pragmatic reasons but it should have had minimal effects on the results concerning the ‘within the sample’ comparisons [25]. In the study people might not have recalled diagnosis of hypertension in the past. It is assumed that the effect of this recall bias is small as diagnosis with hypertension is a major life time event that is less likely to be forgotten. Other limitations of the study were the social desirability bias regarding reporting of treatment among those aware of hypertension and lack of data on other determinants of hypertension such as diabetes and kidney functions.

## Conclusion

Hypertension was common in Uganda but awareness and control was less than optimal as observed elsewhere in sub Saharan Africa [1], [2], [3], [4]. It may be for these reasons that SSA has one of the highest reported incidence and mortality from stroke in the world [9]. Measures for increasing awareness such as screening all adults that get in contact with the health system and at outreach community programs are needed. Because control through treatment is difficult to achieve at population level, optimizing primary preventive approaches that ensures people without hypertension remain in their status and that those with mildly increased blood pressure do not progress to be hypertensive should be the focus for the national policy [16], [19], [27].

## Supporting Information

Table S1 **Percentage distribution of population by age group in the sample compared with the 2002 Uganda population census.**
(DOCX)

## References

[1] Damasceno A, Azevedo A, Siva matos C, Prista A, Diogo D, et al (2009) Hypertension Prevalence, Awareness, treatment, and control in Mozambique (urban/rural gap during epidemiological Transition). Hypertension 54: 77–83. 19470872 10.1161/HYPERTENSIONAHA.109.132423

[2] Addo J, Smeeth L, Leon DA (2007) Hypertension In Sub-Saharan Africa. Hypertension 50: 1012–8. 17954720 10.1161/HYPERTENSIONAHA.107.093336

[3] Agyemang C, Bruijnzeels MA, Owusu-Dabo E (2005) Factors associated with hypertension awareness, treatment, and control in Ghana, West Africa. J Hum Hypertens 20: 67–71. 10.1038/sj.jhh.1001923 16121199

[4] Pereira M, Lunet N, Azevedo A, Barros H (2009) Differences in prevalence, awareness, treatment and control of hypertension between developing and developed countries. J Hypertens (27): 963–975. 10.1097/hjh.0b013e3283282f65 19402221

[5] Wamala JF, Karyabakabo Z, Ndungutse D Guwatudde D (2009) Prevalence factors associated with Hypertension in Rukungiri District, Uganda-A Community-Based Study. Afr Health Sci 9: 153–160. 20589143 PMC2887031

[6] Maher D, Waswa L, Baisley K, Karabarinde A, Unwin N (2011) Epidemiology of hypertension in low-income countries: a cross-sectional population-based survey in rural Uganda. J Hypertens 29: 1061–8. 21505357 10.1097/HJH.0b013e3283466e90

[7] Maher D, Waswa L, Baisley K, Karabarinde A, Unwin N, et al (2011) Distribution of hyperglycaemia and related cardiovascular disease risk factors in low-income countries: a cross-sectional population-based survey in rural Uganda. Int J Epidemiol 40: 160–71. 20926371 10.1093/ije/dyq156 PMC3043279

[8] Walker R, Whiting D, Unwin N, Mugusi F, Swai M, et al (2010) Stroke incidence in rural and urban Tanzania: a prospective, community-based study. Lancet Neurology 9: 786–92. 20609629 10.1016/S1474-4422(10)70144-7

[9] Walker RW, McLarty DG, Kitange HM, Whiting D, Masuki G, et al (2000) Stroke mortality in urban and rural Tanzania. Lancet 355: 1684–7. 10905244 10.1016/s0140-6736(00)02240-6

[10] World Health Organisation. (2009) Global health risks: mortality and burden of disease attributable to selected major risks. Geneva: WHO.

[11] Whelton PK, He J, Muntner P (2000) Prevalence, awareness, treatment and control of hypertension in North America, North Africa and Asia. J Hum Hypertens 18: 545–51. 10.1038/sj.jhh.1001701 15269704

[12] Mayega RW, Makumbi F, Rutebemberwa E, Peterson S, Ostenson CG, et al (2012) Modifiable Socio-Behavioural Factors Associated with Overweight and Hypertension among Persons Aged 35 to 60 Years in Eastern Uganda. PLoS One 7: e47632 doi: 10.1371. 23077653 10.1371/journal.pone.0047632 PMC3471867

[13] World Health Organisation. (2008) STEPS Manual. Geneva: World Health Organisation.

[14] Uganda Bureau of Statistics. (2006) The 2002 Uganda Population and Housing Census, Population Size and Distribution. Kampala: Uganda Bureau of Statistics. Available from http://www.ubos.org/onlinefiles/uploads/ubos/pdf%20documents/2002%20CensusPopnSizeGrowthAnalyticalReport.pdf Accessed 26 December 2012.

[15] Twagiramukiza M, De Bacqueer D, Kips JG, de Backer G, Stichele RV, et al (2011) Current and projected prevalence of arterial hypertension in sub-Saharan Africa by age sex and habitat: an estimate from population studies. J Hypertens 29: 1243–1252. 21540748 10.1097/HJH.0b013e328346995d

[16] World Health Organization. (2004) The global burden of disease. Deaths and DALYs Geneva: World Health Organisation.

[17] Lopez AD, Mathers CD, Ezzati M, Jamison DT, Murray CJL. (2006) Global Burden of Disease and Risk Factors. New York: The World Bank and Oxford University Press. 21250374

[18] Mathers CD, Loncar D (2006) Projections of global mortality and burden of disease from 2002 to 2030. PLOS Med 3(11): e442. 17132052 10.1371/journal.pmed.0030442 PMC1664601

[19] World Health Organization. (2002) World Health Report 2002. Reducing Risks, Promoting Healthy Life. Geneva: World Health Organisation.

[20] Malaza A, Mossong J, Barnighausen T, Newell ML (2012) Hypertension and obesity in adults living in a high HIV prevalence rural area in South Africa. PLoS ONE 7: e47761. 23082211 10.1371/journal.pone.0047761 PMC3474786

[21] Lopez AD, Mathers CD, Ezzati M, Jamison DT, Murray CJ (2006) Global and regional burden of disease and risk factors, 2001: Systematic analysis of population health data. Lancet 367: 1747–1757. 16731270 10.1016/S0140-6736(06)68770-9

[22] World Health Organization. (2004) Cameroon Burden of Diabetes (CamBoD) Project: baseline survey report, Geneva: World Health Organisation.

[23] Tumwesigye E, Wana G, Kasasa S, Muganzi E, Nuwaha F (2010) High uptake of home-based, district-wide, HIV counseling and testing in Uganda. AIDS Patient Care STDS 24: 735–41. 21067357 10.1089/apc.2010.0096

[24] Menzies N, Abang B, Wanyeze R, Nuwaha F, Mugisha B, et al (2009) The costs and effectiveness of four HIV counselling and testing strategies in Uganda. AIDS 23: 395–401. 19114865 10.1097/QAD.0b013e328321e40b

[25] Psaltopoulou T, Orfanos P, Naska A, Lenas D, Trichopoulos D, et al (2004) Prevalence, awareness, treatment and control of hypertension in a general population sample of 26 913 adults in the Greek EPIC study. Int J Epidemiol. 2004 33: 1345–52. 10.1093/ije/dyh249 15218014

[26] Dzudie A, Kengne AP, Muna WF, Ba H, Menanga A, et al (2012) Prevalence, awareness, treatment and control of hypertension in a self-selected sub-Saharan Africa urban population: a cross-sectionsal study. BMJ Open 2: 2012–001217. 10.1136/bmjopen-2012-001217 PMC3433777 22923629

[27] Ministry of Health Uganda. (2010) Health sector strategic and investment plan, Kampala: Ministry of Health, Uganda. Available at: http://www.health.go.ug/docs/HSSIP10.pdf Accessed 26 December 2012.

