# Supplementary material for: Prevalence, Awareness and Control of Hypertension in Uganda
Source: PLoS One. 2013 Apr 17;8(4):e62236. doi: 10.1371/journal.pone.0062236 (PMC3629133; doi:10.1371/journal.pone.0062236)
Supplement: Table S1 — Percentage distribution of population by age group in the sample compared with the 2002 Uganda population census. (DOCX) [file pone.0062236.s001.docx]

**Table S1: Percentage distribution of population by age group in the sample compared with the 2002 Uganda population census**

| Age group | Males | | Females | | Totals | |
| --- | --- | --- | --- | --- | --- | --- |
|  | Sample | Census | Sample | Census | Sample | Census |
| 15-24 | 31.1 | 38.8 | 32.6 | 39.6 | 32.1 | 39.2 |
| 25-34 | 22.9 | 26.0 | 26.9 | 25.7 | 25.4 | 25.9 |
| 35-44 | 16.3 | 14.9 | 17.4 | 14.7 | 17.0 | 15.0 |
| 45-54 | 14.5 | 8.3 | 12.2 | 8.5 | 13.0 | 8.3 |
| 55-64 | 8.0 | 5.7 | 6.2 | 5.6 | 6.8 | 5.5 |
| 65+ | 7.2 | 6.3 | 4.8 | 5.8 | 5.6 | 5.9 |
